# Investigating the microbial community of *Cacopsylla* spp. as potential factor in vector competence of phytoplasma

**DOI:** 10.1111/1462-2920.16138

**Published:** 2022-08-04

**Authors:** Hannes Schuler, Jessica Dittmer, Luigimaria Borruso, Jonas Galli, Stefanie Fischnaller, Gianfranco Anfora, Omar Rota‐Stabelli, Tobias Weil, Katrin Janik

**Affiliations:** ^1^ Faculty of Science and Technology Free University of Bozen‐Bolzano Bozen‐Bolzano Italy; ^2^ Competence Centre for Plant Health Free University of Bozen‐Bolzano Bozen‐Bolzano Italy; ^3^ Université d'Angers, Institut Agro, INRAE, IRHS, SFR Quasav Angers France; ^4^ Department of Forest and Soil Sciences, BOKU University of Natural Resources and Life Sciences Vienna Vienna Austria; ^5^ Research Centre Laimburg Pfatten Italy; ^6^ Research and Innovation Center Fondazione Edmund Mach San Michele all'Adige Italy; ^7^ Center Agriculture Food Environment University of Trento San Michele all'Adige Italy

## Abstract

Phytoplasmas are obligatory intracellular bacteria that colonize the phloem of many plant species and cause hundreds of plant diseases worldwide. In nature, phytoplasmas are primarily transmitted by hemipteran vectors. While all phloem‐feeding insects could in principle transmit phytoplasmas, only a limited number of species have been confirmed as vectors. Knowledge about factors that might determine the vector capacity is currently scarce. Here, we characterized the microbiomes of vector and non‐vector species of apple proliferation (AP) phytoplasma ‘*Candidatus* Phytoplasma mali’ to investigate their potential role in the vector capacity of the host. We performed high‐throughput 16S rRNA metabarcoding of the two principal AP‐vectors *Cacopsylla picta* and *Cacopsylla melanoneura* and eight *Cacopsylla* species, which are not AP‐vectors but co‐occur in apple orchards. The microbiomes of all species are dominated by *Carsonella*, the primary endosymbiont of psyllids and a second uncharacterized Enterobacteriaceae endosymbiont. Each *Cacopsylla* species harboured a species‐specific phylotype of both symbionts. Moreover, we investigated differences between the microbiomes of AP‐vector versus non‐vector species and identified the predominant endosymbionts but also *Wolbachia* and several minor taxa as potential indicator species. Our study highlights the importance of considering the microbiome in future investigations of potential factors influencing host vector competence. We investigated the potential role of symbiotic bacteria in the acquisition and transmission of phytoplasma. By comparing the two main psyillid vector species of Apple proliferation (AP) phytoplasma and eight co‐occurring species, which are not able to vector AP‐phytoplasma, we found differences in the microbial communities of AP‐vector and non‐vector species, which appear to be driven by the predominant symbionts in both vector species and *Wolbachia* and several minor taxa in the non‐vector species. In contrast, infection with AP‐phytoplasma did not affect microbiome composition in both vector species. Our study provides new insights into the endosymbiont diversity of *Cacopsylla* spp. and highlights the importance of considering the microbiome when investigating potential factors influencing host vector competence.

## INTRODUCTION

Phytoplasmas are bacterial pathogens attacking many different crops, trees and ornamental plants, causing hundreds of plant diseases worldwide (Lee et al., 1998; Rao et al., 2018). These plant pathogens are obligate intracellular bacteria that colonize the phloem of different plant species. Transmission between plants is mainly mediated by phloem‐feeding insects, particularly by members of the hemipteran families Cicadellidae, Cixiidae, and Psyllidae (Weintraub & Beanland, 2006). Phytoplasmas are circulative propagative pathogens, that is, they need to be acquired by the insect vector through feeding on infested plants, then the phytoplasma must traverse the gut cells of the insect, replicate, invade the salivary glands, and ultimately reach the saliva to be transmitted to new pserbinlants (Eigenbrode et al., 2017). Therefore, the transmission efficiency of phytoplasmas is a function of the complex association between the insect vector, the host plant, and the phytoplasma itself (Kison & Seemuller, 2001; Perilla‐Henao & Casteel, 2016; Weil et al., 2020).

Apple proliferation (AP) caused by ‘*Candidatus* Phytoplasma mali’ is a severe threat for European apple production (Bertaccini et al., 2014; Janik et al., 2020). An infection with ‘*Ca*. P. mali’ causes the proliferation of axillary shoots (witches' broom) and a decline in fruit size and quality (Bertaccini et al., 2014; Seemüller & Schneider, 2004). It is not possible to cure infected apple trees, and the only way to limit the spread of the disease is to completely remove infected trees from the orchards (Weintraub & Beanland, 2006). ‘*Ca*. P. mali’ is mainly transmitted by the two psyllid species *Cacopsylla picta* (Förster) and *Cacopsylla melanoneura* (Förster) (Carraro et al., 2008; Jarausch et al., 2011, 2019; Tedeschi et al., 2013).

Both species have a similar biology: they are univoltine (i.e. they only complete a single generation per year), overwinter on conifers and return in late winter (*C. melanoneura*) and spring (*C. picta*) to their host plant (i.e. apple), where copulation, oviposition, and immature development take place (Jarausch et al., 2019). *Cacopsylla melanoneura* is oligophagous, feeding on various species of *Malus* and *Crataegus* and occasionally on *Pyrus*, while *C. picta* is strictly monophagous on apple (Jarausch et al., 2019; Ossiannilsson, 1992; Tedeschi et al., 2008). Adults of the new generation leave the host plants in June (*C. melanoneura*) and July (*C. picta*) (Mattedi et al., 2008). Both species co‐occur in most apple‐growing regions of Europe, with some regional exceptions (Jarausch et al., 2019).

Although *Cacopsylla* species were recorded on a wide range of plant hosts, just a few of them are known to act as vectors of phytoplasmas. Apart from the AP‐vectors *C. picta* and *C. melanoneura*, *C. pyri* and *C. pyricola* are vectors of Pear decline caused by ‘*Ca*. P. pyri’, and *C. pruni* is the main vector of European stone fruit yellows caused by ‘*Ca*. P. prunorum’. ‘*Ca*. P. pyri’ and ‘*Ca*. P. prunorum’ are phylogenetically closely related to ‘*Ca*. P. mali’ and also cluster with the 16SrX phytoplasma group (Carraro et al., 1998, 2008; Jarausch et al., 2001). Although phytoplasma presence in several other *Cacopsylla* species has been reported occasionally, none of them was confirmed to transmit the associated pathogens. Therefore, this genus contains both transmitters and non‐transmitters of phytoplasma, but the factors determining vector competence are currently unknown. Especially the fact that, besides the two main AP vectors *C. picta* and *C. melanoneura*, 14 other *Cacopsylla* species have been found in apple orchards (Fischnaller et al., 2017), raises the question which factors influence phytoplasma vector competence in this system.

Factors known to influence the acquisition and virulence of phytoplasma include the plant species, the insect vector, the phytoplasma strain and phytoplasma‐host coevolution (Jarausch & Weintraub, 2013; Orlovskis et al., 2015; Weintraub & Beanland, 2006). Transcriptomic and metabolomics analysis of *C. melanoneura* revealed that infection with AP significantly altered various biological aspects of the host, including specific rhythmic and signalling processes, the actin‐cytoskeleton, the central nervous system and reproduction (Weil et al., 2020). This suggests that AP can alter the vector phenotype and thus increase its transmission. An additional yet understudied factor that might influence the acquisition, replication, and transmission of phytoplasmas is the microbial community of the insect (Alma et al., 2010; Gonella et al., 2019). Bacterial symbionts occupy niches within the host, which are colonized by phytoplasma which may lead to interactions between bacteria and phytoplasma. Endosymbionts are known to influence the ability to acquire and transmit pathogens (Moreira et al., 2009) and therefore the infection with endosymbionts might play an important role in phytoplasma transmission (Gonella et al., 2019).

Primary endosymbionts are ubiquitous in herbivorous hemipteran species (Baumann, 2005; Douglas, 2015; Moran et al., 2005; Sudakaran et al., 2017) and provide essential nutrients lacking in the host's food source (Baumann, 2005; Douglas, 2016). ‘*Candidatus* Carsonella ruddii’, a Gammaproteobacterium, is an essential endosymbiont universally present in psyllid species and harboured in specialized cells (bacteriocytes) forming a bacteriome (Thao, Clark, et al., 2000; Thao, Moran, et al., 2000). With a genome size of 157–174 kb, *Carsonella* has one of the smallest described bacterial genomes, which resulted from genome reduction reflecting the long co‐diversification with its host (Nakabachi et al., 2006; Sloan & Moran, 2012; Tamames et al., 2007). Although most of the genes necessary for the biosynthesis of essential amino acids are present in *Carsonella*, the presence of additional bacteriome‐associated endosymbionts (Buchner, 1965; Fukatsu & Nikoh, 1998) in many psyllids might suggest metabolic complementarity between the two endosymbionts as it has been observed in co‐primary endosymbionts of other hemipterans (Nakabachi et al., 2013; Sloan & Moran, 2012). Additionally, several facultative endosymbionts like *Wolbachia*, *Spiroplasma*, *Rickettsia*, *Sodalis* and *Arsenophonus* have been described in various psyllid species (Ghosh et al., 2020; Morrow et al., 2017; Thao et al., 2001). Some of these symbionts are known to protect their hosts against certain parasites such as viruses, bacteria, eukaryotic parasites and parasitoids (Hedges et al., 2008; Martinez et al., 2019; Moreira et al., 2009; Oliver et al., 2003; Teixeira et al., 2008; Xie et al., 2014; Ye et al., 2013), but their potential interaction with phytoplasma has been poorly investigated.

Recent studies describe the bacterial communities of several *Cacopsylla* species from Japan (Nakabachi et al., 2022), Bhutan (Morrow et al., 2020) and Northwestern United States (Cooper et al., 2017). The microbiome of most *Cacopsylla* species seems to be dominated by several uncharacterized Enterobacteriaceae bacteria (Cooper et al., 2017; Morrow et al., 2020; Nakabachi et al., 2022). Moreover, *Wolbachia* has been described in *C. pruni* (Duron, 2013) and *C. heterogena* (Morrow et al., 2020), a *Sodalis*‐related symbiont in *C. pyri* and *C. burckhardti* (Nakabachi et al., 2022), *Rickettsia* in *C. melanoneura* (Pilgrim et al., 2021) and *Serratia* in *Cacopsylla coccinea* (Nakabachi et al., 2022). Here, we characterized the bacterial community of 10 *Cacopsylla* species occurring in apple orchards by high‐throughput amplicon sequencing of the 16S rRNA gene. To investigate a potential relationship between microbiome composition and host vector capacity, we compared (i) the bacterial communities of phytoplasma‐infected and uninfected individuals of the two AP vector species, and (ii) the bacterial communities of both AP‐vector and non‐vector species present in apple orchards.

## EXPERIMENTAL PROCEDURES

### Insect collection, DNA extraction, and sequencing

Adult insects were collected in various apple orchards in western South Tyrol, Northern Italy, between 2013 and 2017 (Table S1) by beat tray sampling and yellow sticky traps as described by Fischnaller et al. (2017). Insects were sexed, taxonomically identified based on adult morphology according to Ossiannilsson (1992) and Burckhardt (2010) and stored at −80°C. DNA was extracted using the DNeasy Blood & tissue kit (Qiagen, Hilden, Germany) following the instruction of the manufacturer. The presence of phytoplasma was determined using the SYBR‐based qPCR protocol described by Mittelberger et al. (2017).

As a control of DNA quality and to verify taxonomic determination, we amplified the *wingless* (*wg*) gene using the primers LepWG1 and LepWG2 (Brower & DeSalle, 1998). PCR was performed in a total volume of 25 μl using 2.5 μl 10× buffer, 100 μM dNTPs, 0.5 μM of each primer, 0.5 U taq DNA polymerase and 2.5 μl of DNA. PCR cycling conditions were as follows: initial denaturation at 94°C for 3 m; 35 cycles of 94°C for 30 s, 55° for 45 s and 72°C for 45 s followed by a final extension of 72°C for 7 m.

In total, 69 individuals from 10 different species were used for microbial profiling. These included eight phytoplasma‐infected and seven uninfected individuals of *C. picta*, five infected and six uninfected individuals of *C. melanoneura*, seven individuals of *Cacopsylla brunneipennis*, six individuals each of *Cacopsylla breviantennata* and *Cacopsylla affinis*, five individuals each of *Cacopsylla mali*, *Cacopsylla pruni*, *Cacopsylla crataegi* and *Cacopsylla pulchella* and four individuals of *Cacopsylla pyri* (Table S1). Moreover, a no template control (NTC) was included. After amplification of the V4 region of the 16S rRNA gene with barcoded primers 515f and 806r (Caporaso et al., 2017), amplicon sequencing was performed on an Illumina MiSeq with 250 bp paired‐end reads by StarSeq (Mainz, Germany).

### Data analysis

Sequence data were processed in QIIME2 (Bolyen et al., 2019). Forward reads were trimmed at 280 bp and reverse reads at 200 bp. The trimmed reads were quality filtered, denoised, chimera‐checked and clustered into amplicon sequence variants (ASVs) using dada2 (Callahan et al., 2016). Taxonomic classification was performed using the SILVA database v132 (Quast et al., 2013). ASVs identified as archaea, chloroplasts and mitochondria as well as singletons were removed. All ASVs present in the NTC were also removed, except for three ASVs corresponding to *Carsonella* and ‘*Ca*. P. mali’ (Table S2).

For alpha‐ and betadiversity analyses, each sample was rarefied to an even sampling depth of 9001 reads. Bacterial community alpha diversity was determined using the R package ‘vegan’. Bacterial species richness was calculated using the species richness estimator Chao1, bacterial diversity was determined using the Shannon and Simpson diversity indices, and bacterial community evenness was determined using Pielou's evenness index. Differences in alphadiversity indices between species were tested using the Kruskal–Wallis Rank Sum Test followed by pair‐wise Dunn tests. Betadiversity was analysed using principal coordinates analysis (PCoA) based on Bray–Curtis and Jaccard distances in the R package ‘phyloseq’ (McMurdie & Holmes, 2013). Shared or species‐specific genera were visualized using the R package ‘UpsetR’. Differences in the microbial community between vector and non‐vector species were analysed using (i) Random Forest Analysis implemented in the Qiime1.9 script supervised_learning.py and (ii) the Indval function performing Dufrene‐Legendre indicator species analysis from the R package ‘indicspecies’. Both algorithms allow the identification of indicator taxa with enough discriminative power to distinguish bacterial communities. Only taxa with a *p* value < 0.01 and present in at least 50% of individuals were retained as potential indicator taxa. Differences in microbiome composition depending on phytoplasma infection were tested using Adonis and Anosim analyses implemented in Qiime. Importantly, ‘*Ca*. P. mali’ reads were removed from the dataset when testing the impact of phytoplasma infection on microbiome composition. Potential differences due to the sampling year and sampling site were tested using Anosim, Adonis implemented in Qiime and the Envfit function from the R package ‘vegan’.

## RESULTS

### Diverse endosymbiont community within the genus *Cacopsylla*

**FIGURE 1:**
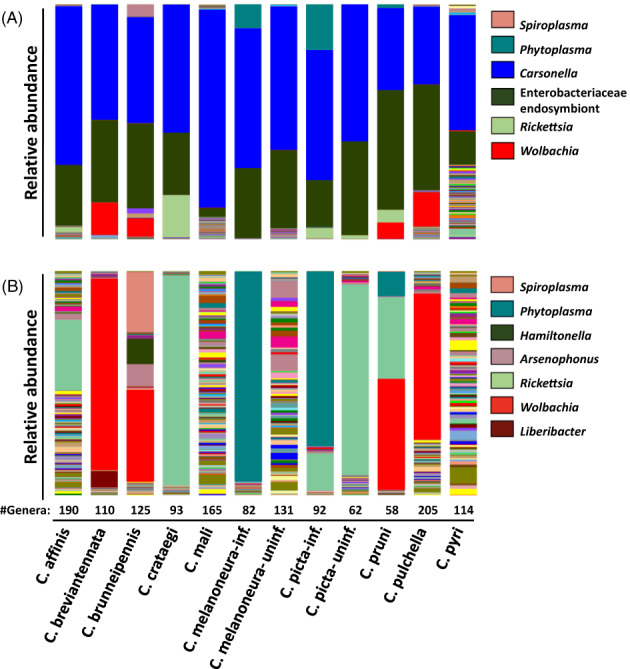
Microbiome composition in 10 *Cacopsylla* species. (A) Shows the entire bacterial community at genus level, dominated by *Carsonella* (blue) and unclassified Enterobacteriaceae endosymbionts (dark green). (B) Removing the dominant primary endosymbionts reveals a high abundance of phytoplasma (cyan), *Rickettsia* (light green) and *Wolbachia* (light blue) in certain species. The total number of bacterial genera per *Cacopsylla* species is provided below the barplot, separating between phytoplasma‐positive (inf) and negative (uninf) individuals of *C. melanoneura* and *C. picta*. The most abundant genera are presented in the legends.

The 16S rRNA gene metabarcoding was performed on 69 individuals from 10 *Cacospylla* species, including the two known vector species of AP‐phytoplasma *C. melanoneura* and *C. picta* and eight non‐AP‐vector species. For the two AP‐vectors, phytoplasma infection status was determined using qPCR to include both infected and uninfected individuals (Table S1). Amplicon sequencing of the V4 region of the 16S rRNA produced on average 177,290 high‐quality reads per individual (range: 9,001–309,108 reads; median: 181,769 reads). These corresponded to 6–338 amplicon sequence variants (ASVs; mean: 102, median: 72) per individual (Table S2). All rarefaction curves reached a plateau, showing that the number of reads was sufficient to capture the bacterial diversity of all individuals (Figure S1). Bacterial taxonomic richness was quite variable between *Cacopsylla* species: While only 58 genera were observed in *C. pruni*, the species *C. pulchella*, *C. affinis* and *C. mali* harboured the most species‐rich bacterial communities with 205, 190 and 165 genera, respectively (Figure 1). Nonetheless, bacterial diversity based on the Shannon index was similar across all species (Figure S2).

**FIGURE 2:**
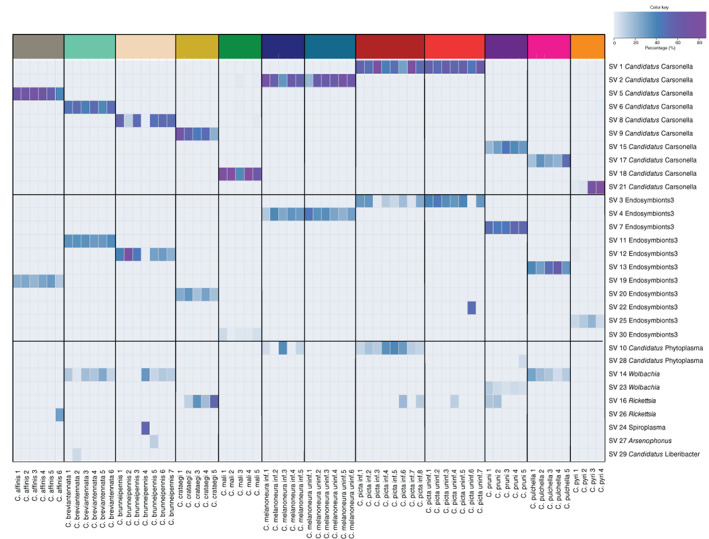
Heatmap showing the distribution of the 30 most abundant amplicon sequence variants (ASVs) in individual psyllids

Despite this high number of bacterial genera, the vast majority of all reads were assigned to only two genera: 53.2% of all reads belonged to the bacteriocyte‐associated primary endosymbiont ‘*Ca*. Carsonella’, whereas 31.7% of the reads belonged to ASVs assigned to unclassified secondary endosymbionts of psyllids from the family Enterobacteriaceae (Figure 1A, Table S2). Specifically, five highly abundant ASVs were identified as either the bacteriome‐associated Y‐symbiont of the mulberry psyllid *Anomoneura mori* (Fukatsu & Nikoh, 1998) or the secondary endosymbiont of *C. myrthi* (Thao, Clark, et al., 2000; Thao, Moran, et al., 2000). Previous studies have shown that these two psyllid endosymbionts are closely related and form a distinct clade among other insect endosymbionts within the Enterobacteriaceae (Morrow et al., 2017, 2020; Thao, Clark, et al., 2000; Thao, Moran, et al., 2000). Moreover, closely related bacteria are known to occur in other *Cacopsylla* species (*C. pyricola*, *C. heterogena* and an unidentified *Cacopsylla* sp. from Bhutan) as well as in other psyllid species from different genera (Morrow et al., 2020). Consistently, the 10 most abundant *Carsonella* ASVs as well as the five ASVs assigned to psyllid secondary endosymbionts showed a high degree of host‐specificity, as each ASV occurred predominantly in a single *Cacopsylla* species (Figure 2). The ASVs related to known psyllid secondary endosymbionts occurred in *C. breviantennata*, *C. crataegi*, *C. melanoneura*, *C. picta*, and *C. pyri*, respectively (Figure 2), whereas five additional abundant ASVs only identified to family level (Enterobacteriaceae) occurred in the remaining species *C. affinis*, *C. brunneipennis*, *C. mali*, *C. pulchella* and *C. pruni*, as well as in a single individual of *C. picta* (Figure 2). These might therefore represent different bacterial taxa.

Besides these two endosymbiont lineages that were present in all individuals of the 10 *Cacopsylla* species, various other ASVs occurred at an abundance of more than 1% of the total reads (Figures 1B and 2). The majority of these were identified as facultative insect endosymbionts or insect‐borne plant pathogens: The Alphaproteobacterium *Wolbachia* was abundant in 20 individuals, including all individuals of *C. breviantennata*, *C. pruni* and *C. pulchella* and four out of seven individuals of *C. brunneipennis*. Notably, the *Wolbachia* harboured by *C. pruni* belonged to a different ASV as the *Wolbachia* harboured by the three other species (Figure 2). *Rickettsia* was found in high abundance (more than 1000 reads per individual) in *C. affinis* (1/5 individuals), *C. crataegi* (4/5 individuals), *C. picta* (3/15 individuals) and *C. pruni* (2/5 individuals) (Figures 1B and 2). Two ASVs were assigned to *Rickettsia*, one of which (SV16) was identical to *Rickettsia* symbionts of other Hemiptera and Coleoptera based on a BLAST search, whereas the second (SV26) was 99.6% identical to the same *Rickettsia* symbionts. The Gammaproteobacterium *Arsenophonus* and the Mollicute *Spiroplasma* were highly abundant in a single individual of *C. brunneipennis* (Figure 2, Figure S3). As expected, ‘*Ca*. P. mali’ was found in all eight individuals of *C. picta* previously tested positive by qPCR (Figure 2). In contrast, ‘*Ca*. P. mali’ was highly abundant in only 3/5 *C. melanoneura* individuals tested positive by qPCR, present at low abundance in one individual and completely absent in the fifth (Figure 2). These two individuals showed the lowest phytoplasma density determined by qPCR, highlighting that a certain phytoplasma density is necessary for its detection by amplicon sequencing (Table S1). In addition, ‘*Ca*. Phytoplasma prunorum’ was highly abundant in a single individual of *C. pruni* (Figure 2). Finally, 10% of the reads of one individual of *C. breviantennata* were assigned to the insect‐borne plant pathogen ‘*Ca*. Liberibacter’. However, a BLAST search revealed that this ASV is identical to ‘*Candidatus* Liberibacter europaeus’, which was described as an endophyte in pears acquired by *C. pyri* (Raddadi et al., 2011).

When considering also low‐abundance ASVs representing less than 1% of the total reads, *Wolbachia* and *Rickettsia* were detected in an even higher number of individuals and species (Table 1). In addition, several ASVs were assigned to (i) bacteriocyte‐associated primary endosymbionts of other Hemiptera (e.g. *Buchnera*, *Sulcia*, *Nasuia*, *Uzinura*) and ants (*Blochmannia*), (ii) gut symbionts of honey bees and bumble bees (*Gilliamella*, *Snodgrassella*, *Commensalibacter*), (iii) various symbionts of protists and amoebae (notably, the intranuclear symbiont ‘*Ca*. Nucleicultrix’ was detected in 19/69 individuals), (iv) arthropod and plant pathogens (e.g. *Pantoea* and *Rhabdochlamydia*, an intracellular pathogen of terrestrial isopods) (Table 1). It is important to note that none of these low‐abundance taxa was detected in the negative control.

**TABLE 1:** Taxa with known symbiotic roles in other systems

		High abundance		Low abundance	
		Individuals	Species	Individuals	Species
Facultative endosymbionts					
*Wolbachia*	Arthropods, nematodes	20/69	4/10	10/69	6/10
*Rickettsia*	Arthopod‐borne pathogens of vertebrates	11/69	4/10	9/69	7/10
*Arsenophonus*	Arthropods, pathogen of strawberry and sugar beet	1/69	1/10	2/69	2/10
*Hamiltonella*	Aphids, whiteflies	1/69	1/10	2/69	2/10
*Spiroplasma*	Various insects, pathogen of citrus and corn	1/69	1/10	1/69	1/10
Bacteriocyte‐associated primary endosymbionts					
*Buchnera*	Aphids	1/69	1/10	6/69	3/10
*Ca*. Sulcia muelleri	Auchenorrhyncha			6/69	4/10
*Ca*. Nasuia	Leafhoppers and treehoppers			3/69	2/10
*Ca*. Blochmannia	Carpenter ants			1/69	1/10
*Ca*. Uzinura	Scale insects			1/69	1/10
Bee gut symbionts					
*Gilliamella*	Honey bees and Bumble bees			3/69	2/10
*Snodgrassella*	Honey bees and Bumble bees			2/69	2/10
*Commensalibacter*	Honey bees and Bumble bees			2/69	2/10
Symbionts of amoebae and protistes					
*Ca*. Nucleicultrix	Intranuclear symbiont of amoebae			19/69	8/10
Holosporaceae	Symbionts of ciliates, diplonemids			7/69	5/10
*Ca*. Protochlamydia	Symbionts of amoebae			4/69	3/10
*Neochlamydia*	Symbionts of amoebae			2/69	2/10
*Ca*. Finniella	Symbionts of amoeboflagellates			1/69	1/10
*Ca*. Megaira	Symbionts of ciliates			1/69	1/10
*Ca*. Paracaedibacter	Symbionts of amoebae			1/69	1/10
*Ca*. Metachlamydia	Parasite of amoebae			1/69	1/10
Arthropod pathogens					
*Rhabdochlamydia*	Intracellular pathogen of terrestrial isopods			2/69	1/10
Insect‐transmitted phytobacteria					
*Pantoea*	Pathogen of maize, transmitted by flea beetle	1/69	1/10	6/69	3/10
*Ca*. Liberibacter	Endophyte of pears transmitted by psyllids	1/69	1/10	1/69	1/10
*Spiroplasma*	Pathogen of citrus and corn, transmitted by leafhoppers	1/69	1/10	1/69	1/10

*Note*: Frequency is provided at individual and species level. The abundance was divided in two categories: high (>1000 reads/individual) and low (<1000 reads/individual).

### Impact of different sampling sites and sampling year on the microbial community of *Cacopsylla* spp.

Psyllids were sampled over several years and from different localities (Table S1). These factors might have influenced microbiome composition to some extent. To address the potential influence of environmental differences between different sites and sampling years, we performed a PCoA based on Bray–Curtis distances on the rarefied ASV table and analysed whether microbiome composition was influenced by (a) psyllid species, (b) sampling year and (c) locality. This was done using Anosim, Adonis and Envfit tests on these three factors. Although both sampling year (Adonis: *R* ^2^ = 0.1, *p* = 0.0001, Anosim: *R* = 0.29, *p* = 0.0001, EnvFit: *R* ^2^ = 0.36, *p* = 0.001) and locality (Adonis: *R* ^2^ = 0.47, *p* = 0.0001, Anosim: *R* = −0.01, *p* = 0.53, EnvFit: *R* ^2^ = 0.39, *p* = 0.014) were significantly correlated with microbiome composition, we found that the factor ‘psyllid species’ had the best fit with the data (Adonis: *R* ^2^ = 0.86, *p* = 0.0001, Anosim: *R* = 0.99, *p* = 0.0001, EnvFit: *R* ^2^ = 0.86, *p* = 0.001; Figure S4). This was most evident for the two AP‐vector species, which were sampled during different years and at different localities, but still clustered tightly by species (Figure S4).

### Microbiome differences between vector‐ and non‐vector species of AP‐phytoplasma

**FIGURE 3:**
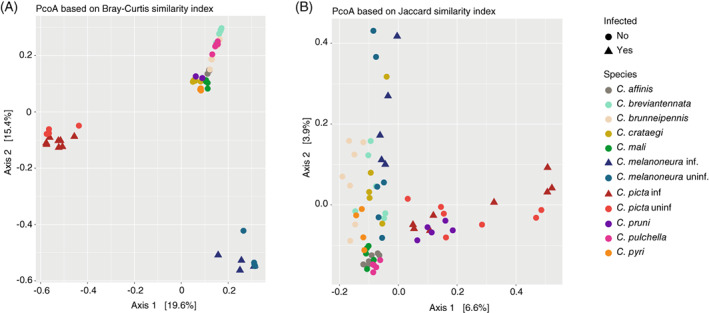
Differences between the microbiomes of AP‐vector and non‐vector species. (A,B) PCoA based on (A) Bray–Curtis distances and (B) Jaccard distances showing differences in microbiome composition between vector and non‐vector species.

Considering that the host species was highly correlated with microbiome composition, we next investigated whether there were significant differences in the microbiomes depending on AP‐vector status. To this end, we performed two PCo analyses: one that considers both taxonomic composition and abundance (Bray–Curtis dissimilarity) and the second based solely on the presence/absence of bacterial taxa (Jaccard dissimilarity). The PCoA based on Bray–Curtis distances revealed three distinct clusters: AP‐infected and uninfected *C. picta* formed one cluster, AP‐infected and uninfected *C. melanoneura* formed another, whereas all eight non‐AP‐vector species formed a third cluster (Figure 3A). However, the PCoA based on Jaccard distances contrasted this pattern and did not show such a clear separation between AP‐vector and non‐AP‐vector species (Figure 3B). Nonetheless, the microbiomes of *C. picta*, *C. pruni* and (to a lesser extent) *C. melanoneura* still diverged to some extent from the other *Cacopsylla* species (Figure 3B).

**FIGURE 4:**
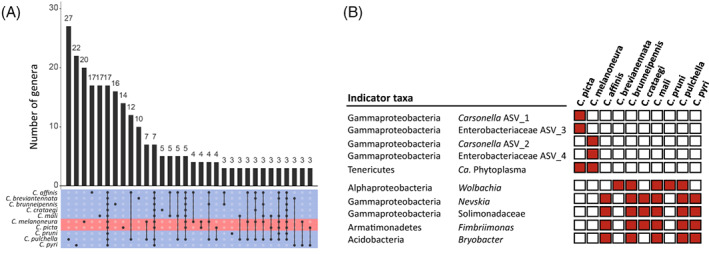
Core microbiome and indicator species identification. (A) Distribution of bacterial genera across *Cacospylla* species. The upper panel shows the number of genera and the lower panel their occurrence in the different species. (B) Distribution of indicator taxa allowing to discriminate between AP‐vector and non‐AP‐vector species. Red squares indicate the detection of a bacterial taxon in at least three individuals of a given psyllid species.

To better understand the potential differences in microbiome composition between the AP‐vector and non‐AP‐vector species, we assessed the distribution of bacterial genera across the psyllid species and found that a large number of genera were unique to a single *Cacopsylla* species, which ranged from 27 genera in *C. pulchella* to three genera in *C. pruni* (Figure 4A). Only 17 genera were shared among all *Cacopsylla* species, and only four among the two AP‐vector species (Figure 4A). Twenty genera were specific for *C. melanoneura* and 14 were specific for *C. picta* (Figure 4A). However, these specific genera occurred at very low abundance in only one to two individuals.

We used Random Forest analysis and Indicator Species analysis to identify ASVs characteristic for *C. picta*, *C. melanoneura* and the eight non‐AP‐vector species. Random Forest Analysis was able to correctly identify the microbiomes of non‐AP‐vector species and of *C. picta*, while only 64% of the *C. melanoneura* samples were correctly identified (Table S3). Nonetheless, both Random Forest and Indicator Species analyses produced concordant results regarding the ASVs with enough discriminative power to distinguish between the three groups. Both analyses identified the species‐specific ASVs of *Carsonella* and the Enterobacteriaceae endosymbiont as the only significant indicator taxa for *C. melanoneura* and *C. picta* (Figure 4B, Table S3). Both analyses also included ‘*Ca*. P. mali’, as it allowed separating both AP‐vector species from the non‐AP‐vector species due to its presence in 50% of the individuals in AP‐vector species.

In addition, Random Forest analysis identified four ASVs as indicator taxa for non‐AP‐vector species. These corresponded to *Wolbachia*, *Nevskia*, the family Solimondaceae and *Fimbriimonas*. Indicator Species Analysis identified the same ASVs as well as an additional one assigned to *Bryobacter* (Figure 4B). These ASVs were indeed absent from the AP‐vector species, but they were not systematically present in all eight non‐AP‐vector species either (Figure 4B). For this purpose, a bacterium was considered ‘present’ in a species when it occurred in at least three individuals of this species. This was based on the consideration that bacteria, which had very few reads in a single individual per species would not be reliable indicators for the species in question. Using this criterion, most indicator taxa were present in six non‐AP‐vector species but absent (or only present in a single individual) in *C. breviantennata* and *C. pruni* (Figure 4B). *Wolbachia* was the only indicator taxon present in these two species.

### Impact of phytoplasma infection on microbiome composition

**FIGURE 5:**
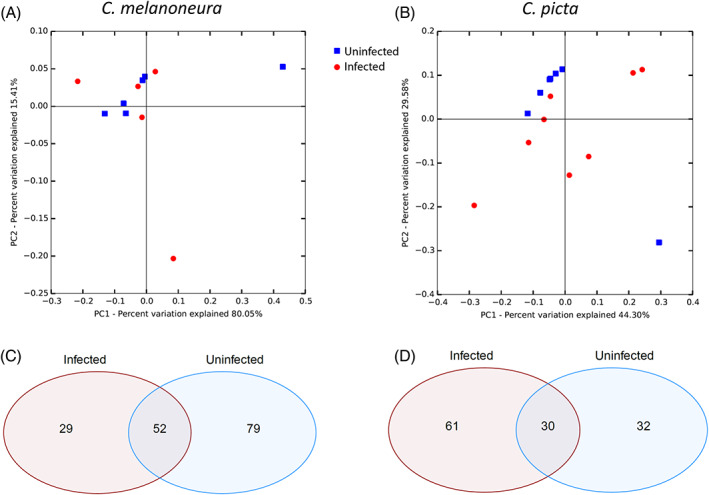
Impact of phytoplasma infection on the microbiomes of *C. melanoneura* (A,C) and *C. picta* (B,D). (A, B) PCo analyses based on Bray–Curtis distances showing the microbiomes of infected versus uninfected individuals. (C, D) Venn diagrams showing the distribution of bacterial genera depending on phytoplasma infection status in each species. Percentages indicate the proportion of reads represented by these genera.

We next investigated whether infection with ‘*Ca*. P. mali’ could be correlated with changes in microbiome composition in the two AP‐vector species *C. melanoneura* and *C. picta*. To this end, all ‘*Ca*. P. mali’ reads were removed from the dataset, to eliminate the presence of phytoplasma as confounding factor. Adonis and Anosim analyses did not detect any significant differences between the microbiomes of infected versus uninfected individuals in either host species (*C. melanoneura*: Adonis: *R* ^2^ = 0.04, *p* = 0.74; Anosim: *R* = −0.04, *p* = 0.62/*C. picta*: Adonis: *R* ^2^ = 0.10, *p* = 0.21; Anosim: *R* = 0.08, *p* = 0.13). Indeed, for *C. melanoneura*, all but one of the infected individuals clustered together with the microbiomes of uninfected individuals, while the microbiomes of uninfected *C. picta* were more homogenous than those of infected individuals (Figure 5A,B). Interestingly, the *C. melanoneura* individual which had a divergent microbiome also had the highest relative abundance of phytoplasma reads. Similarly, the three individuals of *C. picta* with the highest abundance of phytoplasma reads also had the most divergent microbiomes compared to uninfected individuals (Figure S3).

To see whether phytoplasma infection could have an impact on particular bacterial taxa, although not on microbiome composition in general, we also looked at the distribution of bacterial genera between uninfected and infected individuals of both host species. In both species, many bacterial genera were present in either infected or uninfected individuals (Figure 5C,D), at first glance suggesting a correlation between phytoplasma infection status and the presence of certain bacterial taxa. More genera were found in uninfected compared to infected *C. melanoneura*, whereas more genera were detected in infected compared to uninfected *C. picta*. However, these taxa were of low abundance and often occurred in only one to two individuals. The shared taxa on the other hand, represented most of the reads, notably because these included the primary endosymbiont *Carsonella* and the unclassified Enterobacteriaceae endosymbionts, which were highly abundant and present in all individuals. Apart from these two bacterial lineages, the shared taxa contained other highly prevalent genera such as *Acinetobacter* (which was present in all *C. melanoneura* individuals and in 12/15 *C. picta* individuals), *Staphylococcus* (all but one *C. melanoneura* individuals) and *Asinibacterium* (present in nine individuals of each species).

## DISCUSSION

### Potential dual obligatory endosymbiosis in *Cacopsylla* spp.

Most hemipteran species are associated with microbial symbionts that provide essential amino acids lacking in their food source (Baumann, 2005; Douglas, 2016; Sudakaran et al., 2017). Co‐occurring and metabolically complementary primary endosymbionts are known in various hemipteran species such as sharpshooters (McCutcheon & Moran, 2007; Wu et al., 2006), cicadas (McCutcheon et al., 2009), spittlebugs (McCutcheon & Moran, 2010) and mealybugs (McCutcheon & von Dohlen, 2011). In psyllids, a second endosymbiont has been observed to share the bacteriome with *Carsonella* in various species (Fukatsu & Nikoh, 1998; Sloan & Moran, 2012; Subandiyah et al., 2000).

All 10 *Cacopsylla* species characterized in this study harboured highly abundant Enterobacteriaceae endosymbionts in addition to *Carsonella*. Each species not only harboured a species‐specific phylotype of *Carsonella* but also a variant of the second symbiont that was particularly abundant or even unique in this *Cacopsylla* species. Since related bacteria have been observed in several other *Cacopsylla* species (Fukatsu & Nikoh, 1998; Thao, Clark, et al., 2000; Thao, Moran, et al., 2000; Fromont et al., 2016; Cooper et al., 2017; Morrow et al., 2020; Nakabachi et al., 2022; Štarhová Serbina et al. in revision), these could represent a psyllid‐specific endosymbiont clade. The presence of species‐specific genotypes of *Carsonella* and the Enterobacteriaceae endosymbionts is suggestive of an history of co‐divergence with their hosts. In situ localization of both endosymbionts in the bacteriome of *Anomoneura mori* revealed that *Carsonella* was present within the bacteriocytes surrounding the bacteriome, while the second Enterobacteriaceae endosymbiont was present in the syncytium of the bacteriome (Fukatsu & Nikoh, 1998). The same pattern was observed for *Carsonella* and the defensive endosymbiont *Profftella armatura* in the citrus psyllid *Diaphorina citri* (Nakabachi et al., 2013; Subandiyah et al., 2000). Based on these observations, we hypothesize that the Enterobacteriaceae endosymbionts of *Cacopsylla* spp. might also co‐localize with *Carsonella* in the bacteriome. Future studies are needed to clarify the localization, taxonomy and function of these endosymbionts.

### *Rickettsia* and *Wolbachia* are widespread in *Cacopsylla* spp*.*

Apart from the two endosymbionts present in all individuals, we found several facultative symbionts at different frequencies. The most abundant endosymbiont was *Wolbachia*, which has been described in various psyllid genera, including *Diaphorina* (Saha et al., 2012), *Mycopsylla* (Fromont et al., 2016), *Bactericera* (Nachappa et al., 2011), and *Cacopsylla* (Morrow et al., 2020; Shapoval et al., 2021). *Wolbachia* occurred in all individuals of *C. pruni*, *C. breviantennata* and *C. pulchella*, as well as in four out of seven individuals of *C. brunneipennis*. Moreover, low titre infections were found in two individuals of *C. mali*, one individual of *C. pyri* and three individuals of *C. brunneipennis*, highlighting the potential presence of this endosymbiont at low titre in these individuals. The *Wolbachia* belonged to two different ASVs with species‐specific distribution, suggesting that two different *Wolbachia* strains might occur in *Cacopsylla* species from this region. Since the slow evolutionary rate of the 16S rRNA gene prevents an adequate fine‐scale differentiation of different *Wolbachia* strains (Zhou et al., 1998), future studies should focus on a multilocus sequence typing (Baldo et al., 2006) or comparative genomics approach (Gerth & Bleidorn, 2016; Scholz et al., 2020; Wolfe et al., 2021) to resolve the diversity of *Wolbachia* in *Cacopsylla* species. Moreover, specific studies are needed to study the potential phenotypic effect of this symbiont (e.g. Engelstädter & Hurst, 2009).

The second most abundant facultative endosymbiont was *Rickettsia. Rickettsia* is obligatory intracellular bacteria with a wide range of different effects to their hosts (Hurst et al., 1996). Members of this genus are causative agents of diseases in humans, animals and plants, as well as endosymbionts common in various arthropod species (Perlman et al., 2006). In insects, *Rickettsia* is known to cause reproductive effects like male‐killing (Hurst et al., 1996; Werren et al., 1994) and parthenogenesis (Giorgini et al., 2010; Hagimori et al., 2006) but they can also protect their hosts against pathogens (Hendry et al., 2014; Himler et al., 2011). *Rickettsia* is widespread in arthropods where they are estimated to be present in about 24% of terrestrial arthropod species (Weinert et al., 2015) and they have been previously described also in psyllids (Morrow et al., 2020; Pilgrim et al., 2021). Two of the most common ASVs were identified as *Rickettsia*, one of which was found at high frequency in *C. crataegi*, and at lower frequencies in *C. picta* and *C. pruni*. Moreover, this ASV was found at low abundance in one individual of *C. picta*, *C. pruni* and *C. pyri*. The second ASV was found in one individual of *C. affinis* and at low abundance in one individual of *C. melanoneura* and *C. pulchella*. Thus, although *C. melanoneura* has been described to be infected with a Torix group *Rickettsia* (Pilgrim et al., 2021), we detected this symbiont only in a single individual at low abundance. This highlights that more investigations from different geographical areas are needed to obtain a more complete picture of the occurrence of various endosymbionts in these species. Especially the finding that the presence of *Rickettsia* in the midgut of whiteflies had a positive effect on the density of Tomato yellow leaf curl virus and increased its transmission efficiency (Kliot et al., 2014) highlights a potentially important role of this symbiont in pathogen transmission. Since two *C. picta* individuals infected with *Rickettsia* also harboured phytoplasma, it is unlikely that *Rickettsia* hinders phytoplasma acquisition, a potential role in transmission, however, needs to be investigated.

### Presence of rare bacterial taxa as indication for plant‐mediated horizontal transmission

All investigated *Cacopsylla* species were sampled in apple orchards. The shared ecosystem of these psyllids as well as other co‐occurring insects might influence their microbial community. Symbionts can be acquired directly from the plant (Chrostek et al., 2017) as shown for *Cardinium* and *Asaia* in leafhoppers (Gonella et al., 2012, 2015), *Serratia symbiotica* in aphids (Pons et al., 2019a, 2019b), *Hamiltonella defensa* and *Regiella insecticola* in aphids (Henry et al., 2013), *Rickettsia* (Caspi‐Fluger et al., 2011) and *Wolbachia* (Li et al., 2017). Therefore, various insect endosymbionts detected at low prevalence and low abundance, such as *Hamiltonella*, *Pantoea*, *Buchnera*, *Sulcia*, *Nasuia* and *Uzinura*, which are obligatory or facultative endosymbionts of aphids, whiteflies, leafhoppers, treehoppers and scale insects, might represent transient associations. The same applies to symbionts of bumblebees and honeybees, amoebae, protists and ciliates.

Moreover, besides AP‐phytoplasma we found an additional pathogen, namely ‘*Ca*. P. prunorum’, the causal agent of European stone fruit yellows, in one individual of *C. pruni*. ‘*Ca*. Liberibacter’ was also detected in a single individual of *C. breviantennata*, but its 16S rRNA gene sequence was identical to ‘*Ca*. L. europaeus’, known to be a plant endophyte rather than a pathogen (Raddadi et al., 2011). To the best of our knowledge, both species have not been described from apple, suggesting that they were likely acquired from a different source. Although all individuals were sampled in apple orchards, only for *C. picta*, *C. melanoneura* and *C. mali* apple is a known host, that is, a plant on which they can complete their life cycle (Burckhardt et al., 2014; Hodkinson, 2009; Jarausch et al., 2019). Some of the species, including *C. brunneipennis*, *C. crataegi*, *C. pruni and C. pyri*, were found only occasionally in apple orchards (Fischnaller et al., 2017), suggesting that apple might not be considered a suitable host. Gut content analysis of *Cacopsylla* species showed that besides their main host plants they also feed on various occasional food plants (Barthel et al., 2020; Cooper et al., 2019). Hence, different plants (the main host plant and occasional food plants) might act as symbiont reservoirs for each species.

### Similar microbiomes of vector and non‐vector species of AP

We also assessed whether the microbiomes of the two principal AP‐vectors *C. picta* and *C. melanoneura* differ from the microbiomes of non‐AP‐vector species. While we found specific clusters for the two vector species based on Bray–Curtis dissimilarity, which considers the abundance of the bacterial taxa, the differences were less pronounced when using Jaccard dissimilarity to consider only the presence/absence of the bacterial taxa. This indicates that the predominant symbionts *Carsonella* and the Enterobacteriaceae symbiont, which represent the majority of the reads might influence the outcome. This was confirmed by the Random Forest and Indicator Species analyses which showed that only the species‐specific variants of the two major endosymbionts allowed the reliable differentiation of *C. picta* and *C. melanoneura* from non‐AP‐vector species. On the other hand, several taxa occurred only in non‐AP‐vector species and were not observed in AP‐vector species. These included *Wolbachia* and several low‐abundance taxa.

Generally, we found that the microbiomes of the different psyllid species were quite stable in space and time. Various factors such as host species, host developmental stage, the environment, and host diet are known to influence the microbial community (Colman et al., 2012; Douglas, 2015; McLean et al., 2019; Yun et al., 2014). We tested whether different sampling years and localities might have influenced microbiome composition. Although both factors were significantly correlated with microbiome composition, the strongest impact was observed for host species. Especially the fact that different individuals of *C. picta* and *C. melanoneura* were sampled across different years in different localities in South Tyrol, sometimes co‐occurring with non‐AP‐vector species, but still clustered according to species suggests that different environmental conditions and sampling years play a minor role for microbiome composition of the different *Cacopsylla* species. Moreover, since the two AP‐vector species belong to the same mitochondrial clade as the non‐AP‐vector species (Oettl & Schlink, 2015; Percy et al., 2018) and only adults of *Cacopsylla* species were included in the study, a role of the phylogenic relationship and the developmental stage can be excluded.

### Potential influence of endosymbionts on acquisition and transmission of phytoplasma

Although we found a significant difference in the most abundant symbionts of vectors and non‐vector species, we did not identify a single taxon that could act as an indicator species for all eight non‐AP‐vector species. The only taxon at high density is *Wolbachia*, which was absent in both AP‐vector species. *Wolbachia* strains that are naturally present in *Drosophila* fruit flies and artificially transferred to different mosquito species have been shown to reduce the ability of certain pathogens to replicate, thereby inhibiting their transmission (Blagrove et al., 2012; Hedges et al., 2008; Hughes et al., 2011; Moreira et al., 2009; Teixeira et al., 2008). A similar blocking effect against bacteria was observed for *Wolbachia* strains from terrestrial isopods (Braquart‐Varnier et al., 2015) and a competitive exclusion between *Wolbachia* and the acetic acid bacterium *Asaia* has been observed in mosquitoes (Rossi et al., 2015). Although most studies currently focus on vectors of human‐related arboviruses, *Wolbachia* has the potential to control vectors of agricultural diseases as well (Hoffmann et al., 2015; McGraw & O'Neill, 2013; Ross, 2021). *Wolbachia* has been experimentally transferred from the small planthopper *Laodelphax striatellus* to the brown planthopper *Nilaparvata lugens*, the most important vector of stunt virus in rice, which resulted in the rapid invasion of laboratory populations and the reduced transmission of rice tagged stunt virus (Gong et al., 2020). A similar case was described in the planthopper *Dictyophara europaea* where *Wolbachia* was negatively correlated with phytoplasma suggesting that this endosymbiont might have antagonistic effects to the pathogen (Krstić et al., 2018). Although *Wolbachia* was not present in all non‐AP‐vector species, the role of this endosymbiont needs to be investigated in future studies. Artificial transinfection of *C. picta* and *C. melanoneura* should highlight whether *Wolbachia* might inhibit infection and acquisition of phytoplasmas similar to other pathosystems (Gong et al., 2020; Hoffmann et al., 2015; McGraw & O'Neill, 2013). Population genetic surveys are needed to study the presence of *Wolbachia* in different populations and confront it with the presence of phytoplasma in natural field populations.

### The role of phytoplasma influencing the microbial community of *Cacopsylla* spp.

Acquisition and transmission of phytoplasma requires the ingestion of phytoplasma by feeding on the phloem of an infected host plant, it must then invade the intestinal tract, enter the gut, and traverse the basal lamina to enter the hemolymph where they then move to the salivary glands and are introduced back into the plant by feeding on the phloem (Hogenhout et al., 2008). Thus, phytoplasmas colonize niches that are occupied by bacterial symbionts and therefore might interact with them (Gonella et al., 2019). The presence of phytoplasma might therefore have a direct impact on the microbial community of the host. This has been shown in insect vectors of ‘*Candidatus* Phytoplasma solani’ where individuals of *Euscelis incises* and *Euscelidius variegatus* infected with phytoplasma had significantly higher alphadiversity than uninfected individuals (Moussa et al., 2020). In contrast, the microbial community of other vector species such as *Cicadella viridis*, *Dictyophara europaea*, *Hyalesthes obsoletus*, *Phylaenus spumarius* and *Psammotettix spp*. did not change significantly. This is in line with our study, since we found no significant effect of phytoplasma infection on the microbial community of *C. melanoneura* and *C. picta*. However, the individuals with the most phytoplasma reads seemed to have more divergent microbiomes. While the microbiomes of infected *C. picta* were more heterogeneous and contained some low‐abundance taxa that were not observed in uninfected individuals, infected *C. melanoneura* harboured more low‐abundance taxa than uninfected individuals. This suggests that there may be differences between the two AP‐vector species that should be further investigated.

## CONCLUSION

Here, we studied the microbial community of 10 *Cacopsylla* species from apple orchards in Northern Italy. Each psyllid species harboured species‐specific phylotypes of the two major endosymbionts, ‘*Ca*. Carsonella ruddii’ and uncharacterized Enterobacteriaceae, suggesting the presence of two obligate endosymbionts in *Cacopsylla* species. We found differences between the microbiomes of vector and non‐vector species of Apple proliferation phytoplasma, which were mostly due to the predominant endosymbionts of the vector species and *Wolbachia* as well as several minor taxa in non‐AP‐vector species. Notably, the presence of at least two different *Wolbachia* strains in various non‐AP‐vector species opens an interesting new research avenue investigating whether this endosymbiont might influence the vector competence of these insect species.

## CONFLICT OF INTEREST

The author declares that there is no conflict of interest that could be perceived as prejudicing the impartiality of the research reported.

## Supporting information


**Suppl. Table S1** Sampling information and phytoplasma infection status for each of the 69 individuals.

**Suppl. Table S2** ASV table.

**Suppl. Table S3** Random Forest and Indval results.

**Suppl. Figure S1** Rarefaction curve of all 69 individuals.

**Suppl. Figure S2** Bacterial diversity of *Cacopsylla spp*. based on richness (Chao 1), Shannon and Simpson diversity indices, and Pielou's evenness index.

**Suppl. Figure S3** Microbiome composition in each individual sample showing the entire bacterial community at genus level, dominated by *Carsonella* (blue) and unclassified Enterobacteriaceae endosymbionts (dark green).

**Figure S4** Supplementary Figure.

**Suppl. Document S1** List of statistical codes used in the study.

## Data Availability

Raw sequence data will be deposited in public databases after manuscript acceptance.
