# Supplementary figures and images for: Investigating the microbial community of Cacopsylla spp. as potential factor in vector competence of phytoplasma
Source: Environ Microbiol. 2022 Aug 4;24(10):4771–86. doi: 10.1111/1462-2920.16138 (PMC9804460; doi:10.1111/1462-2920.16138)

**Rarefaction curves of all samples**

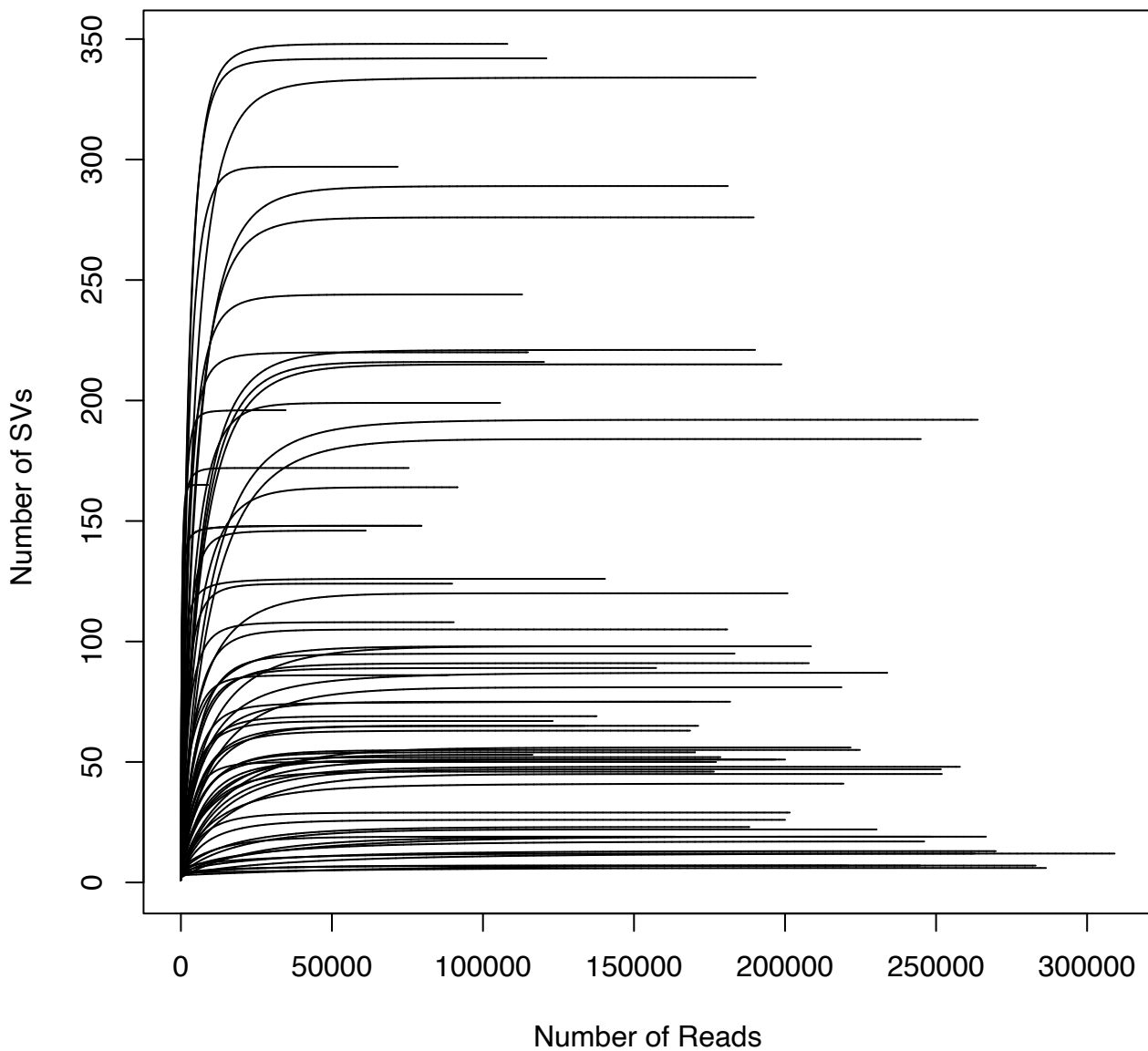

Supplement: Supplementary file 4 — Suppl. Figure S1 Rarefaction curve of all 69 individuals. [file EMI-24-4771-s002.pdf]

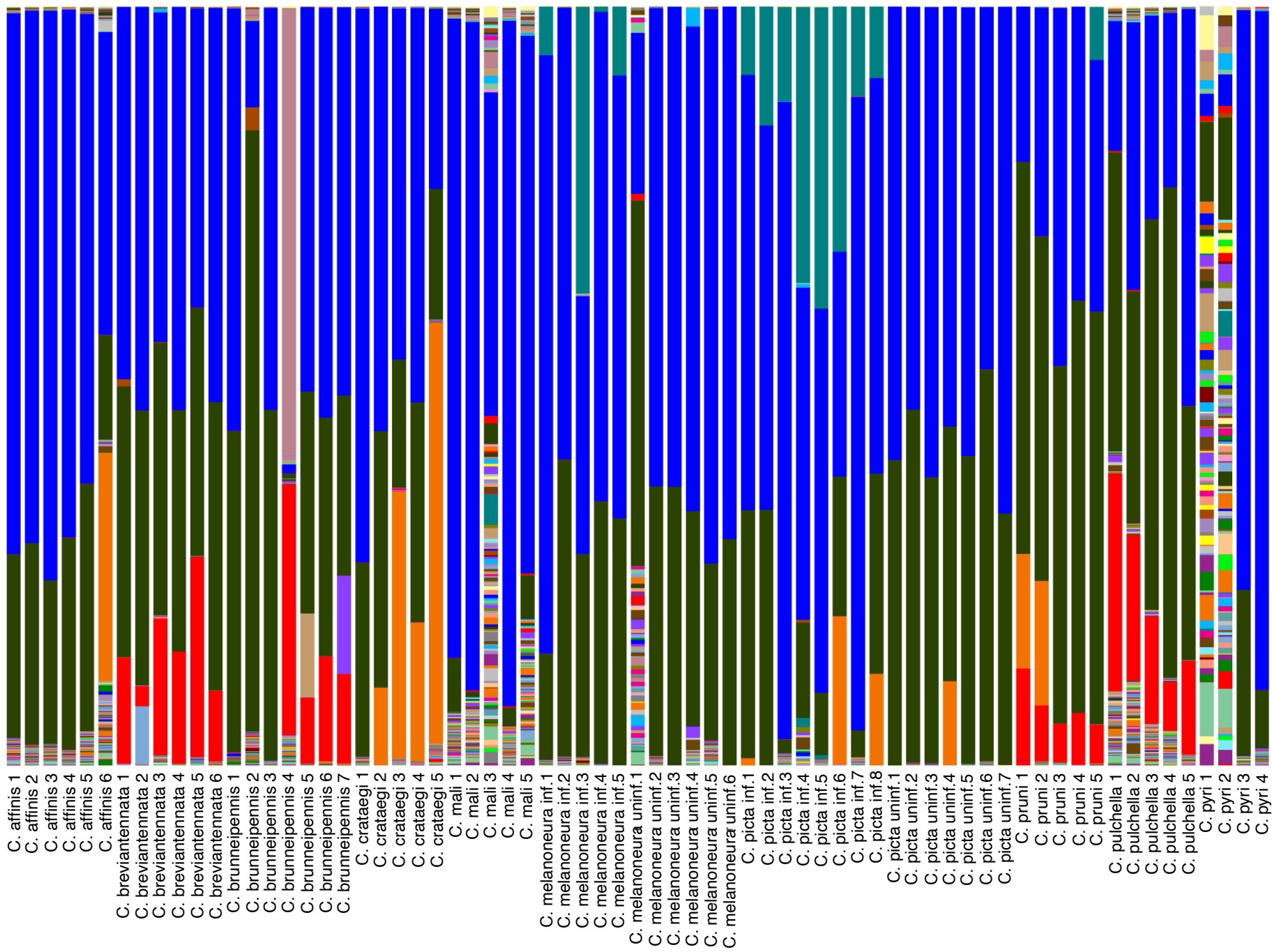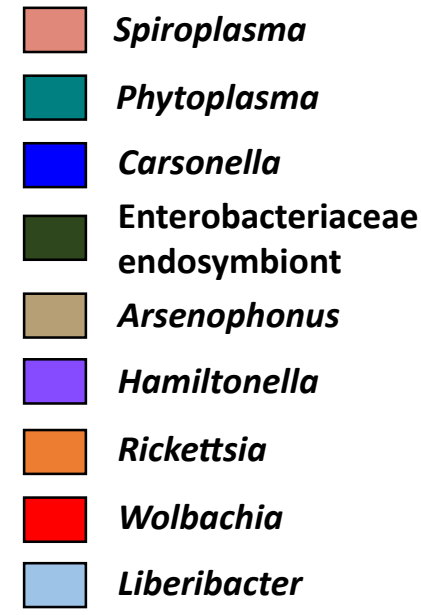

Supplement: Supplementary file 6 — Suppl. Figure S3 Microbiome composition in each individual sample showing the entire bacterial community at genus level, dominated by Carsonella (blue) and unclassified Enterobacteriaceae endosymbionts (dark green). [file EMI-24-4771-s007.pdf]
