# Supplementary material for: Investigating the microbial community of Cacopsylla spp. as potential factor in vector competence of phytoplasma
Source: Environ Microbiol. 2022 Aug 4;24(10):4771–86. doi: 10.1111/1462-2920.16138 (PMC9804460; doi:10.1111/1462-2920.16138)
Supplement: Supplementary file 5 — Suppl. Figure S2 Bacterial diversity of Cacopsylla spp. based on richness (Chao 1), Shannon and Simpson diversity indices, and Pielou's evenness index. [file EMI-24-4771-s005.pdf]

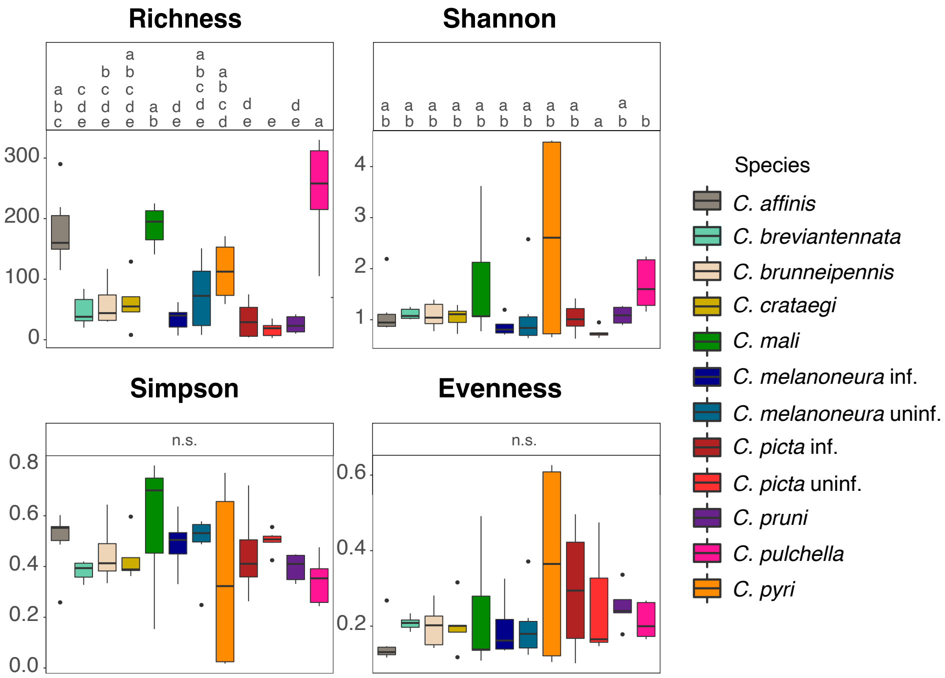
Richness
Shannon
Simpson
Evenness
n.s.
n.s.
Species
C. affinis
C. breviantennata
C. brunneipennis
C. crataegi
C. mali
C. melanoneura inf.
C. melanoneura uninf.
C. picta inf.
C. picta uninf.
C. pruni
C. pulchella
C. pyri
