# Supplementary material for: Investigating the microbial community of Cacopsylla spp. as potential factor in vector competence of phytoplasma
Source: Environ Microbiol. 2022 Aug 4;24(10):4771–86. doi: 10.1111/1462-2920.16138 (PMC9804460; doi:10.1111/1462-2920.16138)
Supplement: Supplementary file 7 — Figure S4 Supplementary Figure. [file EMI-24-4771-s008.pdf]

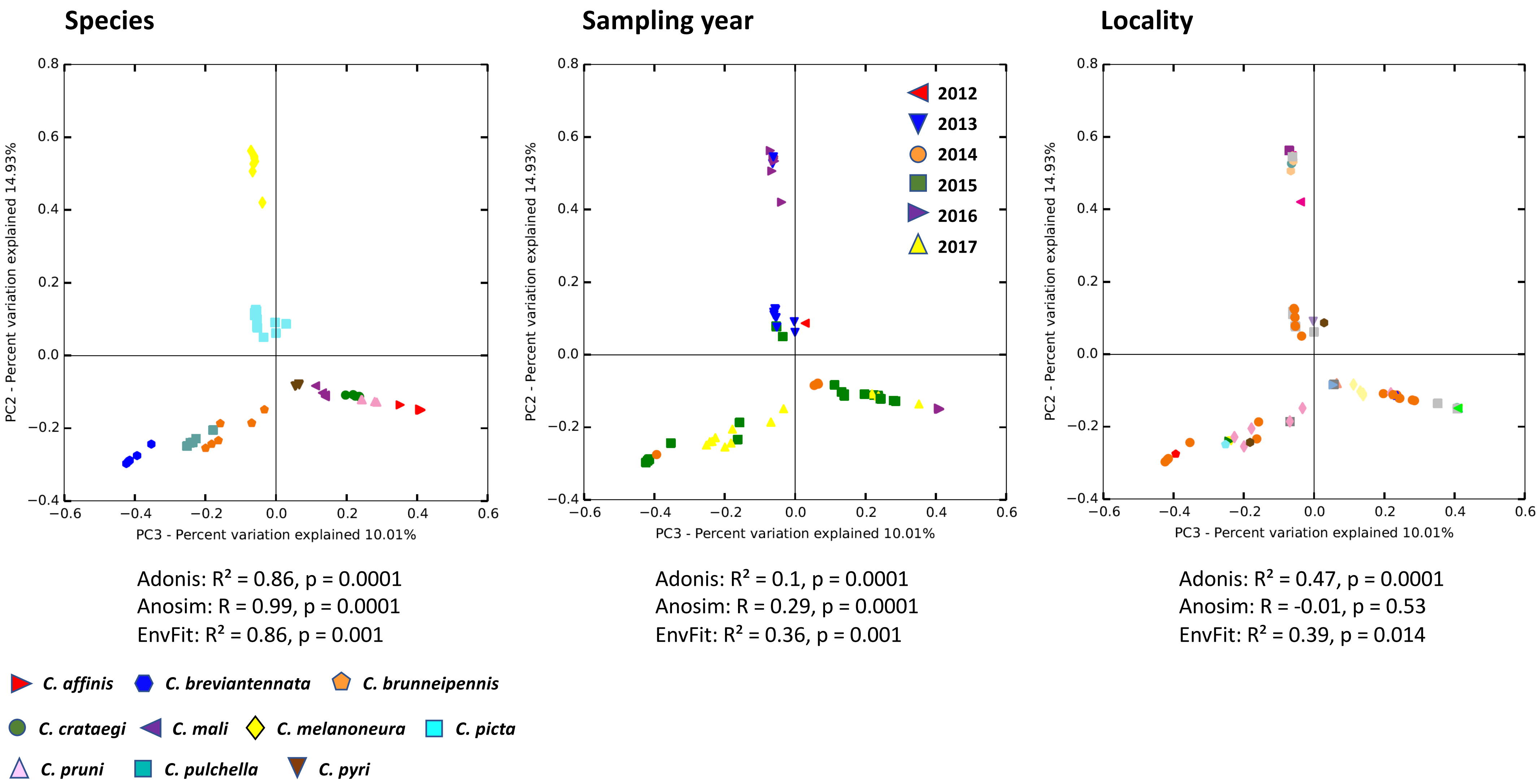
Species
Sampling year
Locality
2012
2013
2014
2015
2016
2017
PC2 - Percent variation explained 14.93%
PC3 - Percent variation explained 10.01%
Adonis: R² = 0.86, p = 0.0001
Anosim: R = 0.99, p = 0.0001
EnvFit: R² = 0.86, p = 0.001
Adonis: R² = 0.1, p = 0.0001
Anosim: R = 0.29, p = 0.0001
EnvFit: R² = 0.36, p = 0.001
Adonis: R² = 0.47, p = 0.0001
Anosim: R = -0.01, p = 0.53
EnvFit: R² = 0.39, p = 0.014
C. affinis
C. breviantennata
C. brunneipennis
C. crataegi
C. mali
C. melanoneura
C. picta
C. pruni
C. pulchella
C. pyri
